# NUP62 promotes breast cancer progression and inhibits ferroptosis by stabilizing NRF2 in a KEAP1-dependent way

**DOI:** 10.1016/j.isci.2026.115484

**Published:** 2026-03-26

**Authors:** Ziran Qiu, Yu Lin, Shanzheng Chen, Wenqing Cao, Jun Yang, Rui Li, Yuan Chen, Xiaoqing Yao, Senlin Fu, Na Jin

**Affiliations:** 1 Department of Breast and Thyroid Surgery, Loudi Central Hospital, No. 51, Changqing Middle Street, Loudi City, Hunan Province, China; 2 Department of Radiation Oncology, National Cancer Center/National Clinical Research Center for Cancer/Cancer Hospital, Chinese Academy of Medical Sciences and Peking Union Medical College, Beijing, China

**Keywords:** pharmacology, molecular biology, cancer

## Abstract

Identification of drug resistance drivers of breast cancer (BC) is a multifaceted challenge. Here, we demonstrated that NUP62 is significantly upregulated in BC tissues and cell lines, and its high expression correlates with a poor prognosis. Functional experiments revealed that NUP62 promotes BC cell proliferation, migration, and malignant phenotypes, while inhibiting ferroptosis. Mechanistically, NUP62 competitively binds to KEAP1, disrupting KEAP1-mediated ubiquitination and degradation of NRF2. This stabilization promotes NRF2 nuclear translocation, enhancing the transcription of antioxidant genes and inhibiting ferroptosis. Crucially, eribulin—identified through virtual screening of FDA-approved compounds—selectively inhibited NUP62, destabilizing NRF2 and abrogating its nuclear localization. *In vivo*, eribulin or NUP62 silencing significantly suppressed tumor growth in xenograft models. Our findings establish the NUP62-KEAP1-NRF2 axis as a master regulator of ferroptosis in BC, positioning eribulin as a promising therapeutic agent for NUP62-high tumors.

## Highlights

• NUP62 is upregulated in breast cancer and correlates with a poor prognosis
• NUP62 competitively binds KEAP1 to stabilize NRF2 and suppress ferroptosis
• NUP62 knockdown enhances lipid peroxidation and induces ferroptosis in BC
• Eribulin targets NUP62 to destabilize NRF2 and inhibit tumor growth *in vivo*

## Graphical abstract

**Figure:**
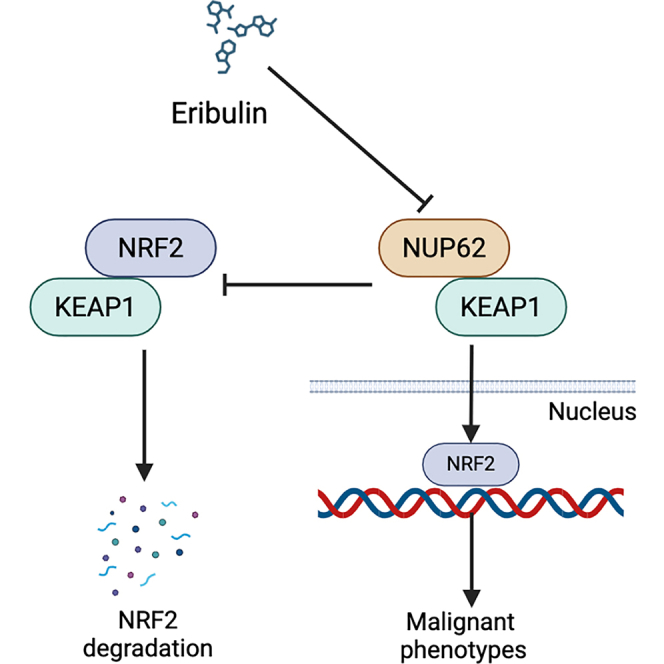

## Introduction

Breast cancer (BC) remains the most common malignancy and second leading cause of cancer mortality in women worldwide.^1^ While early detection and therapeutic strategies have advanced significantly, recurrence and metastatic progression continue to compromise patient survival.^2^ This clinical challenge is compounded by significant metastatic heterogeneity driven by genetic diversity, epigenetic dysregulation, and tumor microenvironmental interactions.^3^ Further mechanistic studies are required to identify novel molecular determinants and therapeutic targets for improved intervention.

The nuclear pore complex (NPC) serves as the exclusive gateway for molecular exchange between the nuclear and cytoplasmic compartments.^4^ Composed of approximately 30 distinct nucleoporins (NUPs), the NPC features FG-repeat domains in approximately one-third of its constituent NUPs (e.g., NUP62).^5^ These intrinsically disordered FG-repeat regions form a dynamic, hydrogel-like meshwork that mediates selective nucleocytoplasmic transport.^6^ NUPs perform non-canonical roles beyond the NPC, directly modulating gene expression and chromatin dynamics in the nuclear and cytoplasmic compartments, thereby regulating developmental progression.^7^ NUP62, located in the central avenue of the NPC, plays a vital role in regulating selective transport between the nucleus and cytoplasm.^8^

Ferroptosis is an iron-dependent cell death modality driven by lethal lipid peroxidation, exhibiting distinct morphological and mechanistic features from apoptosis.^9^ Targeting ferroptosis has demonstrated promise as a therapeutic strategy against cancers, yet multiple defense mechanisms—notably NRF2 activation—confer resistance to this cell death modality.^10^ KEAP1 serves as the primary negative regulator of NRF2. Under basal conditions, NRF2 is sequestered in the cytoplasm by KEAP1 and targeted for ubiquitin-proteasomal degradation, maintaining its inactivity.^11^ Upon exposure to reactive oxygen species (ROS) or electrophilic insults, KEAP1-mediated degradation is inhibited, facilitating NRF2 dissociation and nuclear translocation. Nuclear NRF2 forms heterodimers with small musculoaponeurotic fibrosarcoma proteins (MAF) proteins to activate antioxidant response element (ARE)-driven transcription of cytoprotective genes, thereby suppressing ferroptosis.^12^ Given the pivotal role of redox homeostasis in regulating ferroptosis sensitivity, NRF2 functions as a central regulator of this process. Thus, targeting the KEAP1-NRF2 axis represents a promising therapeutic strategy to overcome ferroptosis resistance and enhance susceptibility to ferroptosis in BC.

This study explores the role of NUP62 in BC. NUP62 is upregulated in BC tissues and cell lines, correlating with a poor prognosis. It promotes BC cell proliferation/migration and inhibits ferroptosis by competitively binding KEAP1 to stabilize NRF2, blocking its ubiquitination. Eribulin, identified as a NUP62 inhibitor, destabilizes NRF2 and suppresses BC growth, which establishes the NUP62-KEAP1-NRF2 axis as a key target for BC precision therapy.

## Results

### NUP62 expression is upregulated in BC and correlates with a poor prognosis

**Figure 1:**
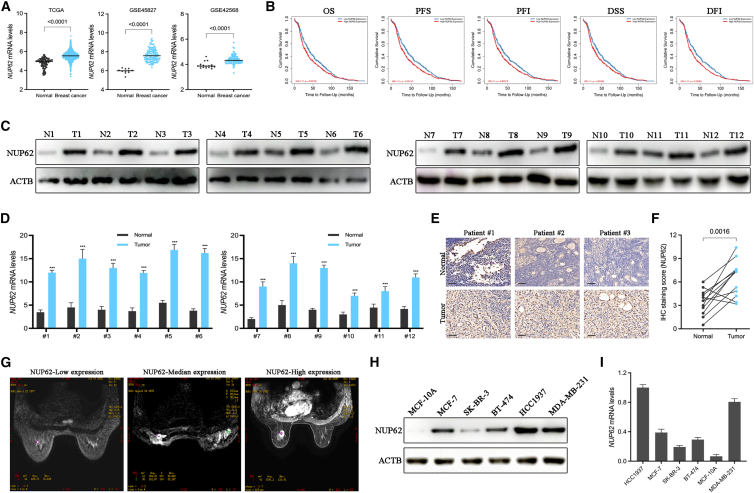
NUP62 expression is upregulated in BC and correlates with a poor prognosis (A) NUP62 mRNA levels in BC samples compared to non-cancerous samples by TCGA and GEO database. (B) Survival analysis of NUP62 in BC cohorts based on TIMER database. (C) NUP62 protein levels in 12 BC tumor tissues and paired normal tissues were detected using western blotting. (D) NUP62 mRNA levels in 12 BC tumor tissues and paired normal tissues were detected using RT-qPCR. (E) Representative IHC images of NUP62 protein levels in BC tumor tissues and normal tissues. Scale bars, 150 μm. (F) NUP62 expression was plotted based on the immunohistochemical score. (G) Representative MRI images of BC patients. (H) NUP62 protein expression was detected in human breast epithelial cell (MCF-10A) and various BC cell lines by using western blotting. (I) NUP62 mRNA expression was detected in human breast epithelial cell (MCF-10A) and various BC cell lines by using RT-qPCR. Data are presented as the mean ± SD. ∗*p* < 0.05, ∗∗*p* < 0.01, ∗∗∗*p* < 0.001.

Given the poorly characterized expression patterns and mechanisms of NUP62 in BC, we retrieved transcriptomics and clinical data from TCGA and GEO databases. Bioinformatic analysis demonstrated upregulated NUP62 expression in BC (Figure 1A), which correlated significantly with reduced overall survival (OS), progression-free survival (PFS), progression-free interval (PFI), disease-specific survival (DSS), and disease-free interval (DFI) (Figure 1B). Subsequent collection of BC patient tissues at our clinical center followed by western blot and qPCR analysis revealed broad NUP62 expression in tumor tissues, with levels significantly elevated compared with those in paired adjacent normal tissues (Figures 1C and 1D). Immunohistochemistry (IHC) staining also showed the significantly higher expression of NUP62 in BC tissues compared with adjacent normal tissues (Figures 1E and 1F). MRI images of three typical BC patients with low, median, and high expression of NUP62 are shown in Figure 1G; the breast tumors of the patients with high NUP62 expression were found to be larger. We further verified NUP62 mRNA and protein levels in BC cell lines (Figures 1H and 1I). These results indicate that the expression of NUP62 in BC is increased compared with normal breast tissues, which is closely related to the poor prognosis of BC patients.

### NUP62 induces malignant phenotypes of BC cells

**Figure 2:**
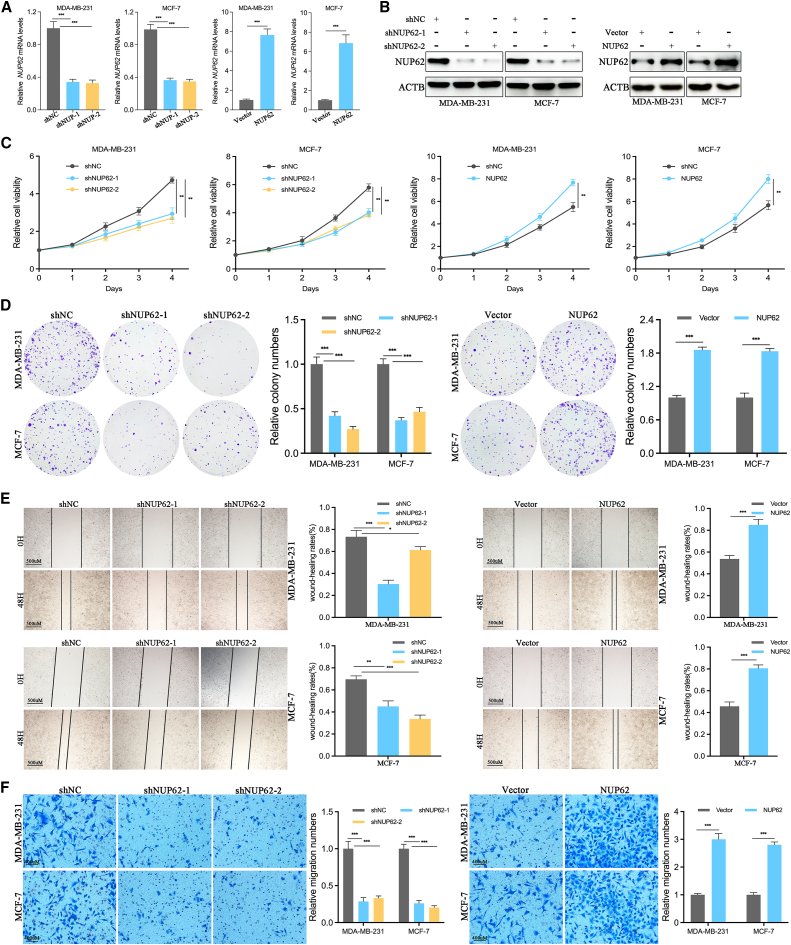
NUP62 promotes breast cancer cell malignant progression *in vitro* (A) The overexpression and knockdown efficiencies of NUP62, assessed via RT-qPCR. (B) The overexpression and knockdown efficiencies of NUP62, assessed via western blotting. (C) CCK-8 assays were performed to test the effect of NUP62 overexpression or knockdown on BC cell viability. (D) Colony-formation assays were performed to test the effect of NUP62 overexpression or knockdown on BC cell proliferation. (E) Wound-healing assays were performed to test the effect of NUP62 overexpression or knockdown on BC cell migration. Scale bars, 500 μm. (F) Transwell assays were performed to test the effect of NUP62 overexpression or knockdown on BC cells migration. Scale bars, 400 μm. Data are presented as the mean ± SD. ∗*p* < 0.05, ∗∗*p* < 0.01, ∗∗∗*p* < 0.001.

As shown in Figures 2A and 2B, shRNAs or OE-plasmids could significantly decrease or increase NUP62 expression in BC cells. NUP62 knockdown significantly suppressed BC cell proliferation, and NUP62 overexpression increased BC cell proliferation in CCK-8 assays and colony-formation assays (Figures 2C and 2D). Wound-healing assays revealed that NUP62 knockdown suppressed the motility of BC cells, and NUP62 overexpression increased BC cell motility (Figure 2E). Transwell assays also confirmed the above results (Figure 2F). Taken together, these results confirmed that NUP62 acts as an oncogene in BC.

### NUP62 disrupts redox homoeostasis and inhibits ferroptosis in BC cells

**Figure 3:**
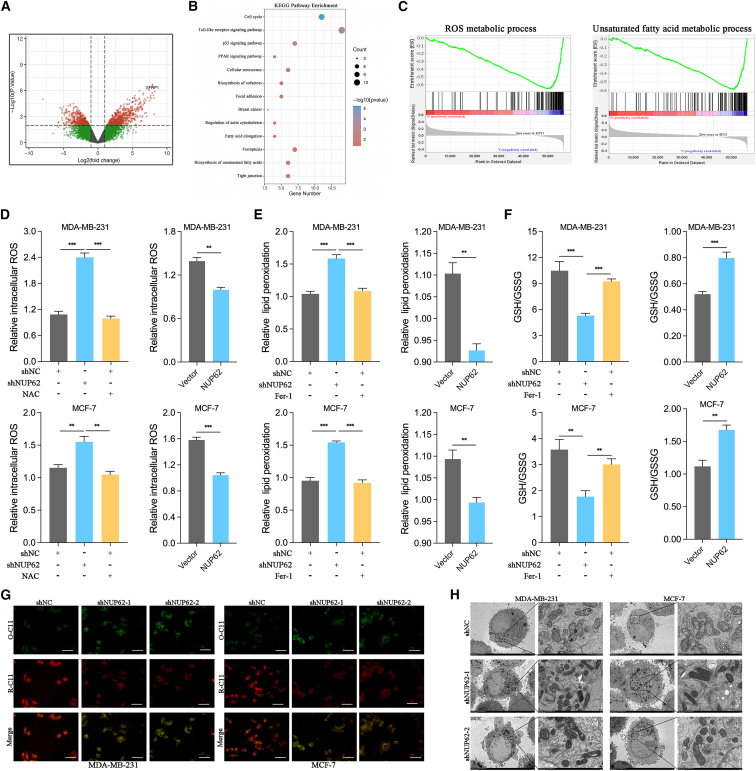
NUP62 disrupts redox homoeostasis and inhibits ferroptosis in BC cells (A) Volcano plots displaying significant DEGs in MCF-7 cells. (B) KEGG analysis of the top 13 pathways regulated by NUP62. (C) GSEA shows that the ROS metabolic process and unsaturated fatty acid metabolic process pathways were enriched. (D) The levels of intracellular ROS, quantified in indicated BC cells. (E) The levels of lipid peroxidation, measured in indicated BC cells. (F) The GSH/GSSG ratio, measured in indicated BC cells. (G) BODIPY 581/591 C11 was used to detect lipid peroxidation in indicated BC cells. Scale bars, 40 μm. (H) Representative images of mitochondrial morphology, shown by TEM, in indicated BC cells. Data are presented as the mean ± SD. ∗*p* < 0.05; ∗∗*p* < 0.01; ∗∗∗*p* < 0.001.

To investigate the molecular mechanism underlying NUP62-mediated BC progression, we conducted RNA-seq to identify differentially expressed genes (DEGs) in MCF-7 cells with stable NUP62 knockdown and controls (Figure 3A). Compared with controls, NUP62 knockdown induced 853 upregulated and 684 downregulated DEGs. KEGG pathway analysis of these DEGs demonstrated significant enrichment in ferroptosis pathways, such as the biosynthesis of unsaturated fatty acids and fatty acid elongation (Figure 3B). Gene set enrichment analysis (GSEA) revealed that ROS and unsaturated fatty acid metabolic processes were activated in the low-NUP62 expression group (Figure 3C). These findings indicate that NUP62 participates in the regulation of redox homeostasis and ferroptosis.

Increased lipid peroxidation and elevated ROS are hallmarks of ferroptosis. We next investigated the role of NUP62 in redox homeostasis. NUP62 knockdown significantly elevated the total ROS levels compared with controls in BC cells, an effect that could be reversed by the antioxidant N-acetyl-L-cysteine (NAC, 5 mM for 24 h). Conversely, NUP62 overexpression markedly reduced the total ROS levels in BC cells versus controls (Figure 3D). Ferroptosis is initiated by redox imbalance, with lipid ROS and the GSH/GSSG ratio emerging as well-established biomarkers. As shown in Figures 3E and 3F, NUP62 knockdown markedly elevated the lipid peroxidation levels and decreased the GSH/GSSG ratio compared with controls, effects abolished by the ferroptosis inhibitor Fer-1 (10 μM for 24 h). In contrast, NUP62 overexpression in BC cells markedly reduced lipid peroxidation levels and elevated the GSH/GSSG ratio compared with controls.

To further investigate whether NUP62 knockdown increases cell ferroptosis, we determined lipid peroxidation by C11-BODIPY581/591 staining. The results showed that knockdown of NUP62 resulted in excessive lipid peroxidation (Figure 3G). In addition, transmission electron microscopy (TEM) was performed to confirm the occurrence of ferroptosis. As shown in Figure 3H, shNUP62-knockdown BC cells showed shrinkage of mitochondria and mitochondrial membrane rupture, accompanied by an increase in membrane density and ridge reduction or even disappearance. Collectively, these results indicate that NUP62 regulates redox homeostasis and ferroptosis.

### NUP62 stabilizes NRF2 protein by decreasing its degradation

**Figure 4:**
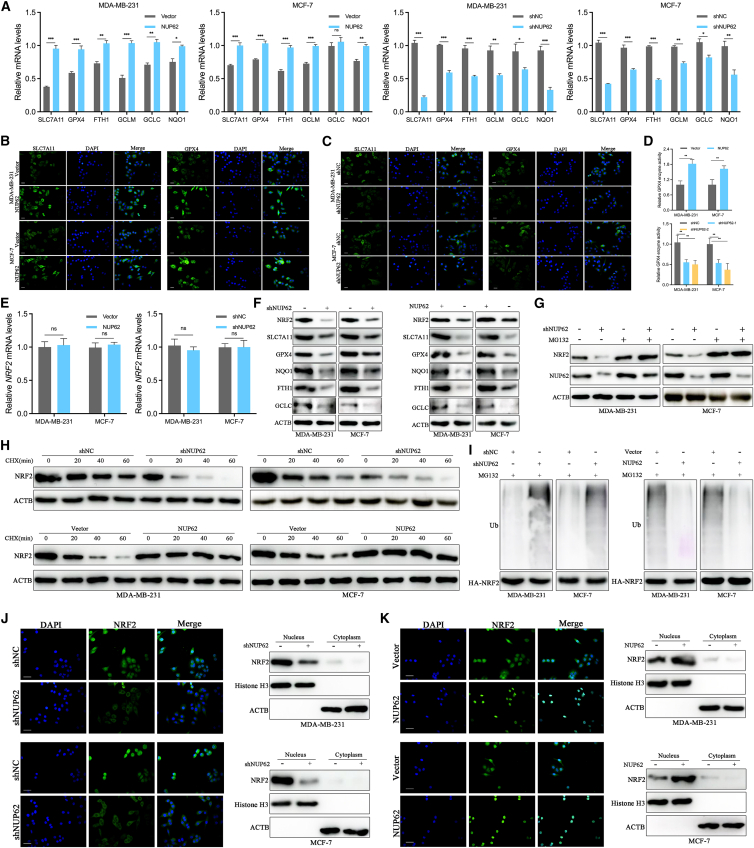
NUP62 stabilizes the NRF2 protein by decreasing its degradation (A) The expression levels of antioxidant and ferroptosis-related genes were quantified by RT-qPCR in indicated BC cells. (B) SLC7A11 and GPX4 protein expressions were measured by IF staining in BC cells with NUP62 overexpression. Scale bars, 10 μm. (C) SLC7A11 and GPX4 protein expressions were measured by IF staining in BC cells with NUP62 knockdown. Scale bars, 10 μm. (D) GPX4 enzyme activity was measured in BC cells after NUP62 knockdown or overexpression. (E) mRNA levels of NRF2 in the BC cells with NUP62 knockdown or overexpression. (F) Protein levels of NRF2, SLC7A11, and GPX4 in the BC cells with NUP62 knockdown or overexpression. (G) BC cells with NUP62 knockdown or controls were treated with MG132 (10 μM) for 4 h, and NRF2 protein levels were analyzed by western blotting. (H) NRF2 protein half-life of BC cells with NUP62 overexpression or knockdown was evaluated using western blotting. (I) NRF2 ubiquitination levels of BC cells with NUP62 overexpression or knockdown. (J) NUP62 knockdown inhibited NRF2 nuclear translocation in BC cells, as evidenced by IF microscopy and western blotting. Scale bars, 10 μm. (K) NUP62 overexpression facilitated NRF2 nuclear translocation in BC cells, as evidenced by IF microscopy and western blotting. Scale bars, 10 μm. Data are presented as the mean ± SD. ∗*p* < 0.05, ∗∗*p* < 0.01, ∗∗∗*p* < 0.001.

To investigate the mechanisms by which NUP62 regulates redox homeostasis and ferroptosis, we analyzed the expression of antioxidant and ferroptosis-related genes in NUP62-overexpressing or -knockdown cells. NUP62 knockdown decreased mRNA levels of these genes, whereas overexpression significantly upregulated their expression in BC cells (Figure 4A). Furthermore, the decreased expression of SLC7A11 and GPX4 induced by NUP62 knockdown was validated by immunofluorescence (IF) staining in BC cells, but NUP62 overexpression resulted in significant increases in SLC7A11 and GPX4 protein expression (Figures 4B and 4C). We further explored whether NUP62 affected the GPX4 enzyme activity. The results showed that NUP62 knockdown inhibited GPX4 enzyme activity, while NUP62 overexpression enhanced GPX4 enzyme activity in BC cells (Figure 4D).

Given that NRF2 is a well-established key regulator of ferroptosis and antioxidant response, and our preliminary observations in BC cells indicated a potential link between NUP62 depletion and altered NRF2 pathway activity, we examined whether NUP62 regulates NRF2 signaling. As presented in Figures 4E and 4F, NUP62 overexpression and knockdown did not affect NRF2 mRNA levels but, respectively, upregulated and downregulated NRF2 protein levels, implying that NUP62 may regulate NRF2 stability at the post-transcriptional level. This was further validated by MG132 treatment (10 μM for 4 h), which restored decreased NRF2 protein expression in NUP62-silenced BC cells (Figure 4G). Next, we examined the degradation rate of NRF2 and observed that NRF2 protein exhibited accelerated degradation in NUP62-silenced cells but displayed enhanced stability in NUP62-overexpressing cells (Figure 4H). Consistently, NUP62 silencing significantly elevated NRF2 ubiquitination, whereas NUP62 overexpression markedly decreased it (Figure 4I). We next investigated the impact of NUP62 on NRF2 nuclear translocation. Both western blot and IF analyses revealed that the nuclear NRF2 protein levels were significantly decreased by NUP62 silencing and increased by NUP62 overexpression (Figures 4J and 4K).

### NUP62 competes with NRF2 for KEAP1 binding

**Figure 5:**
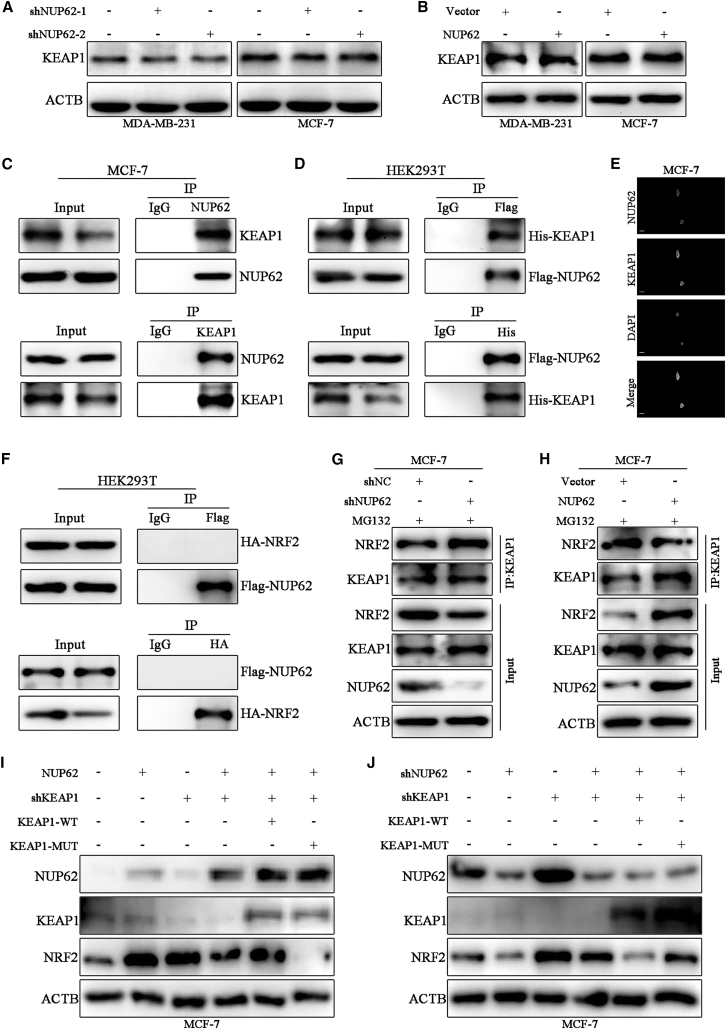
NUP62 competes with NRF2 for KEAP1 binding in BC cells (A) KEAP1 protein expression was examined in BC cells after NUP62 knockdown via western blot analysis. (B) KEAP1 protein expression was examined in BC cells after NUP62 overexpression via western blot analysis. (C) CoIP assay of endogenous NUP62 and KEAP1 binding in MCF-7 cells. (D) CoIP assay of exogenous NUP62 and KEAP1 binding in HEK293T cells. (E) The localization of endogenous NUP62 and KEAP1 in MCF-7 cells was determined by immunofluorescence. Scale bars, 15 μm. (F) CoIP assay of exogenous NUP62 and NRF2 binding in HEK293T cells. (G) MCF-7 cells with NUP62 knockdown were treated with MG132 and cell lysates underwent IP using KEAP1 antibody, followed by western blot analysis with NRF2 antibodies. (H) MCF-7 cell with NUP62 overexpression was treated with MG132 and cell lysates underwent IP using KEAP1 antibody, followed by western blot analysis with NRF2 antibodies. (I and J) Western blot showing NUP62, KEAP1, and NRF2 expressions in MCF-7 cells with indicated treatments. Data are presented as the mean ± SD. ∗*p* < 0.05, ∗∗*p* < 0.01, ∗∗∗*p* < 0.001.

Given KEAP1’s established role as a central regulator of NRF2 ubiquitination and proteasomal degradation, we hypothesized that NUP62-induced NRF2 stabilization might be mechanistically linked to its interaction with KEAP1. We first examined the impact of NUP62 knockdown or overexpression on KEAP1 expression and observed that NUP62 exerted no effect on KEAP1 protein levels (Figures 5A and 5B). Therefore, we investigated whether NUP62 physically interacts with KEAP1 to inhibit NRF2 degradation. CoIP assays revealed that endogenous NUP62 exhibited binding to endogenous KEAP1 (Figure 5C), which was confirmed using exogenous Flag-NUP62 and His-KEAP1 in HEK293T cells (Figure 5D). Moreover, IF staining confirmed that NUP62 and KEAP1 predominantly co-localized in the nucleus of MCF-7 cells (Figure 5E). However, coIP assay showed that Flag-NUP62 did not interact with HA-NRF2 in HEK293T cells (Figure 5F). Next, we investigated the impact of NUP62 silencing or overexpression on NRF2-KEAP1 binding. As depicted in Figures 5G and 5H, NUP62-silenced cells exhibited increased NRF2-KEAP1 binding, whereas this binding was diminished in NUP62-overexpressing cells. To further confirm that the regulation of NUP62 on NRF2 depends on KEAP1, we used KEAP1 knockout or mutant (R483S) that cannot interact with NRF2 to demonstrate the role of KEAP1 in mediating the regulation of NUP62 on NRF2. The results showed that NUP62 overexpression increased and NUP62 knockdown decreased NRF2 levels, which was weakened after KEAP1 knockdown. Reintroduction of KEAP1-WT, but not KEAP1-MUT, reversed NRF2 expression changes (Figures 5I and 5J). Collectively, these results demonstrated that NUP62 competes with NRF2 for binding to KEAP1, thereby disrupting the stability of the KEAP1-NRF2 complex and promoting subsequent NRF2 degradation.

### NUP62 drives progression and ferroptosis inhibition of BC via regulation of NRF2

**Figure 6:**
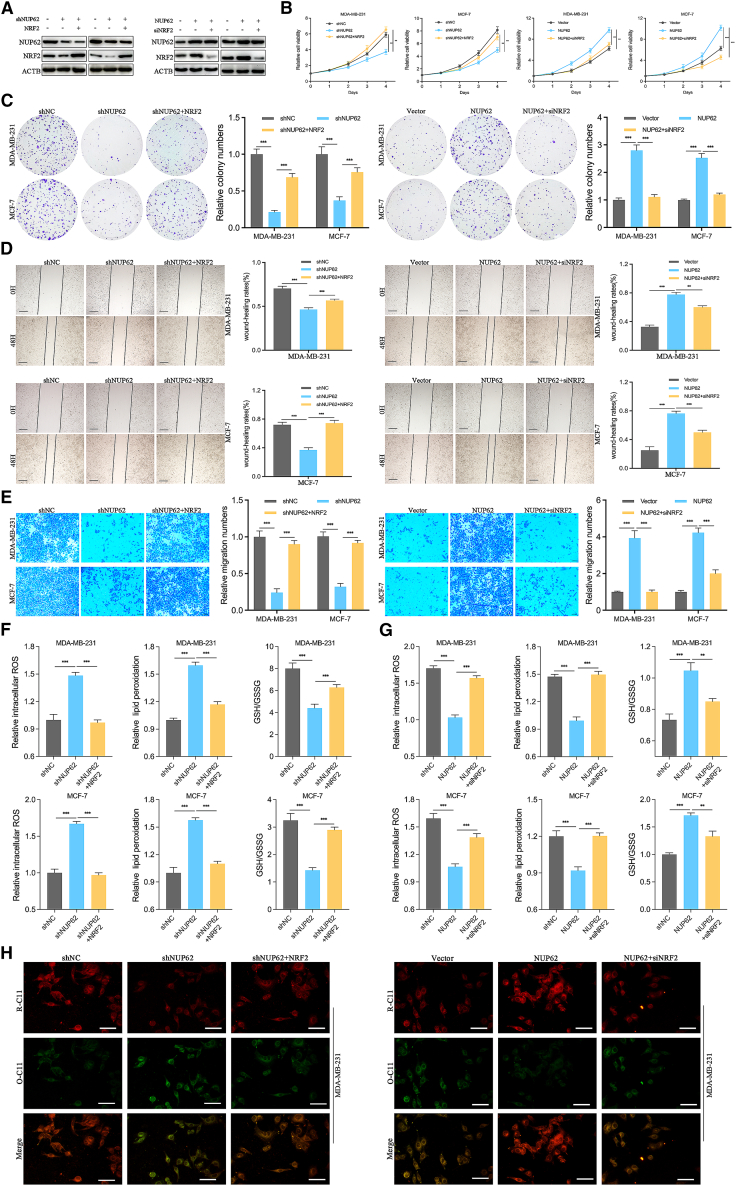
NUP62 drives progression and ferroptosis suppression of BC cells via regulation of NRF2 (A) BC cells with NUP62 knockdown or overexpression were transfected with NRF2 plasmids or NRF2-siRNA, respectively, and the expression of NUP62 or NRF2 was measured using western blot analysis. (B) Cell viability was assessed by CCK-8 assays in NUP62-knockdown or -overexpressing BC cells transfected with NRF2 plasmids or NRF2-siRNA, respectively. (C) Cell proliferation was assessed by colony-formation assays in NUP62-knockdown or NUP62-overexpressing BC cells transfected with NRF2 plasmids or NRF2-siRNA, respectively. (D) Cell migration was assessed by wound-healing assays in NUP62-knockdown or NUP62-overexpressing BC cells transfected with NRF2 plasmids or NRF2-siRNA, respectively. Scale bars, 500 μm. (E) Cell migration was assessed by Transwell assays in NUP62-knockdown or NUP62-overexpressing BC cells transfected with NRF2 plasmids or NRF2-siRNA, respectively. Scale bars, 200 μm. (F) Intracellular ROS, lipid peroxidation, and GSH/GSSG ratio were measured in NUP62-knockdown BC cells transfected with NRF2 plasmids. (G) Intracellular ROS, lipid peroxidation, and GSH/GSSG ratio were measured in NUP62-overexpressing BC cells transfected with NRF2-siRNA. (H) BODIPY 581/591 C11 was used to detect lipid peroxidation in NUP62-knockdown or NUP62-overexpressing BC cells transfected with NRF2 plasmids or NRF2-siRNA, respectively. Scale bars, 40 μm. Data are presented as the mean ± SD. ∗*p* < 0.05, ∗∗*p* < 0.01, ∗∗∗*p* < 0.001.

To further investigate the role of NRF2 in NUP62-induced BC progression, we modulated NRF2 expression by transfecting NUP62-overexpressing cells with NRF2-siRNA and NUP62-knockdown cells with NRF2-overexpression plasmid (Figure 6A). Overexpression of NRF2 abolished the inhibitory effects on cell growth and migration by NUP62 knockdown in BC cells. Conversely, NRF2 inhibition attenuated the promoting effects on cell growth and migration by NUP62 overexpression in BC cells (Figures 6B–6E). In addition, overexpression of NRF2 blocked the increase in lipid peroxidation levels and decrease in GSH/GSSG ratio in NUP62-knockdown BC cells (Figure 6F). In contrast, NRF2 inhibition reversed the decrease in lipid peroxidation levels and increase in GSH/GSSG ratio in NUP62-overexpressing BC cells (Figure 6G). We further determined lipid peroxidation by C11-BODIPY581/591 staining. The results showed that the overexpression of NRF2 rescued the increased production of excessive lipid peroxidation by NUP62 knockdown in MDA-MB-231 cells, while NRF2 inhibition reversed the decreased production of excessive lipid peroxidation by NUP62 overexpression in MDA-MB-231 cells (Figure 6H). These results suggest that NUP62 promotes BC progression and inhibits ferroptosis in a NRF2-dependent manner.

### Eribulin is a potential inhibitor of NUP62

**Figure 7:**
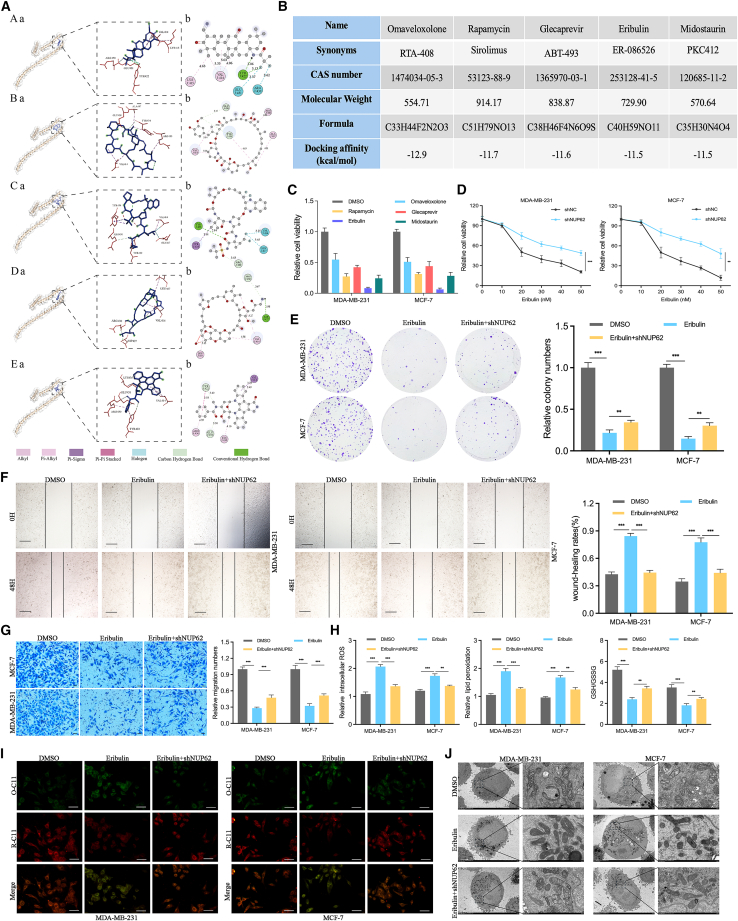
Eribulin is a novel inhibitor of NUP62 (A) 3D and 2D structures of NUP62 protein in complex with omaveloxolone, rapamycin, glecaprevir, eribulin, and midostaurin. (B) Basic information of five compounds, omaveloxolone, rapamycin, glecaprevir, eribulin, and midostaurin. (C) Cell viability was assessed by CCK-8 assays in BC cells treated with omaveloxolone, rapamycin, glecaprevir, eribulin, and midostaurin. (D) Cell viability was assessed by CCK-8 assays in eribulin-treated BC cells with or without NUP62 knockdown. (E) Cell proliferation was assessed by colony-formation assays in eribulin-treated BC cells with or without NUP62 knockdown. (F) Cell migration was assessed by wound-healing assays in eribulin-treated BC cells with or without NUP62 knockdown. Scale bars, 500 μm. (G) Cell migration was assessed by Transwell assays in eribulin-treated BC cells with or without NUP62 knockdown. Scale bars, 400 μm. (H) Intracellular ROS, lipid peroxidation, and GSH/GSSG ratio were measured in eribulin-treated BC cells with or without NUP62 knockdown. (I) BODIPY 581/591 C11 was used to detect lipid peroxidation in eribulin-treated BC cells with or without NUP62 knockdown. Scale bars, 40 μm. (J) Representative images of mitochondrial morphology, shown by TEM, in eribulin-treated BC cells with or without NUP62 knockdown. Scale bars, 2 μm (left) and 500 nm (right). Data are presented as the mean ± SD. ∗*p* < 0.05, ∗∗*p* < 0.01, ∗∗∗*p* < 0.001.

To discover potential NUP62 inhibitors, we performed virtual molecular docking screening of an FDA-approved drug library comprising 1,618 compounds. Based on the binding affinity, we finally selected the top five candidates—omaveloxolone, rapamycin, glecaprevir, eribulin, and midostaurin for further analysis, which were validated to exhibit anticancer activity^13^^,^^14^^,^^15^^,^^16^ (Figures 7A and 7B). BC cells were incubated with each compound (0.5 μM) for 24 h. Cell activity was measured, and eribulin (>80% inhibitory effect) was selected for subsequent investigation (Figure 7C). Next, we examined the effect of eribulin on the malignant phenotypes of BC cells. We found that eribulin significantly inhibited the proliferative and migratory abilities, and when NUP62 was knocked down, eribulin activity was partly abolished, as evidenced by CCK-8, colony-formation, wound-healing and Transwell assays (Figures 7D–7G). Similar trends were observed for the lipid peroxidation levels, GSH/GSSG ratio, C11-BODIPY581/591 staining, and TEM results (Figures 7H–7J). These results suggest eribulin as a potential inhibitor of NUP62.

### Eribulin destabilizes NRF2 protein and inhibits its nuclear localization

**Figure 8:**
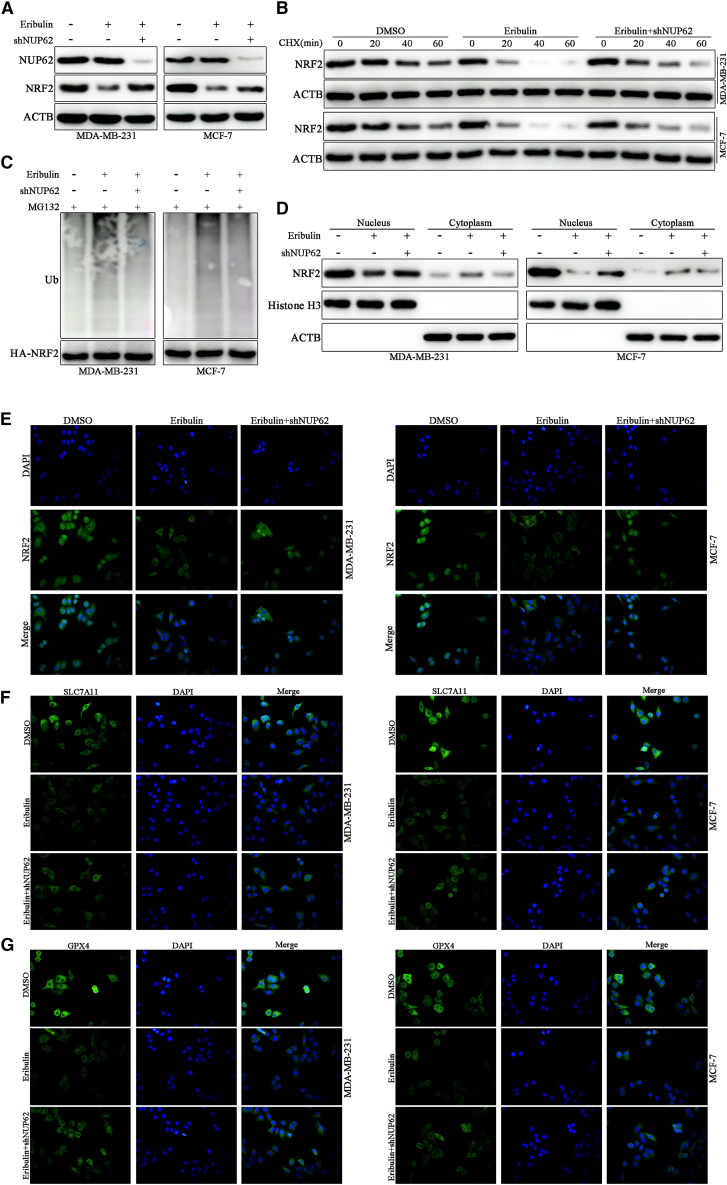
Eribulin destabilizes NRF2 protein and inhibits its nuclear localization (A) The protein levels of NRF2 in eribulin-treated BC cells with or without NUP62 knockdown. (B) NRF2 protein half-life of eribulin-treated BC cells with or without NUP62 knockdown, evaluated using western blotting. (C) NRF2 ubiquitination levels of eribulin-treated BC cells with or without NUP62 knockdown. (D) Nucleus-cytoplasm distribution of NUP62 in indicated BC cells, as evidenced by western blotting. (E) Nucleus-cytoplasm distribution of NUP62 in indicated BC cells, as evidenced by IF microscopy. (F) SLC7A11 protein expression , measured by IF staining in indicated BC cells. (G) GPX4 protein expression, measured by IF staining in indicated BC cells. Data are presented as the mean ± SD. ∗*p* < 0.05, ∗∗*p* < 0.01, ∗∗∗*p* < 0.001.

We further investigated whether eribulin modulates NRF2 protein expression through a mechanism analogous to NUP62 knockdown. We treated BC cells with eribulin and found that eribulin induced a significant reduction in the NRF2 protein level, while showing no effect on NUP62 protein expression (Figure 8A). Interestingly, eribulin-mediated suppression of NRF2 protein expression was markedly abrogated after NUP62 was knocked down (Figure 8A), confirming the dependency of eribulin’s inhibitory effect on NUP62. To elucidate whether eribulin suppresses NRF2 protein stability through inhibition of NUP62-mediated deubiquitination, we conducted half-time and ubiquitination assays. Eribulin treatment markedly decreased NRF2 protein stability and increased its ubiquitination levels; however, these effects were partially abolished in NUP62-knockdown cells (Figures 8B and 8C). Similarly, eribulin significantly reduced the protein abundance of NRF2 in the nucleus of BC cells, and this effect was partially alleviated after NUP62 knockdown (Figures 8D and 8E), further confirming the dependency of eribulin’s regulatory action on NUP62. Furthermore, the decreased expression of SLC7A11 and GPX4 proteins induced by eribulin was restored in NUP62-knockdown BC cells (Figures 8F and 8G). These results suggest that eribulin destabilizes NRF2 protein and inhibits its nuclear localization in a NUP62-dependent manner.

### Eribulin or NUP62 knockdown inhibits tumor growth *in vivo*

**Figure 9:**
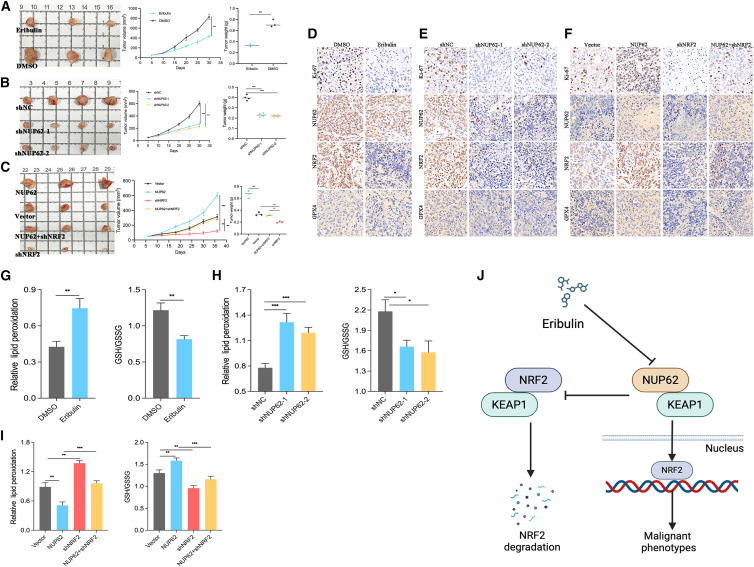
Eribulin or NUP62 knockdown inhibits tumor growth *in vivo* (A) The volume and size of subcutaneous tumors in mice with or without eribulin treatment. (B) The volume and size of subcutaneous tumors in mice with NUP62 knockdown. (C) The volume and size of subcutaneous tumors in mice after NRF2 knockdown in NUP62-overexpressing MDA-MB-231 cells. (D–F) Representative IHC staining images of Ki-67, NUP62, NRF2, and GPX4 in tumor tissue sections of each group. (G–I) Lipid peroxidation and GSH/GSSG ratio were measured in tumor tissue of each group. (J) A schematic model showing that NUP62 protects BC cells against ferroptosis by sustaining NRF2 signaling activation. Data are presented as the mean ± SD. ∗*p* < 0.05, ∗∗*p* < 0.01, ∗∗∗*p* < 0.001.

To determine whether eribulin or NUP62 silencing inhibits tumorigenesis of BC *in vivo*, we implanted the indicated MDA-MB-231 cells into nude mice. The results demonstrated that eribulin or NUP62 silencing significantly suppressed tumor growth, evidenced by reduced tumor volumes and tumor weights (Figures 9A and 9B). IHC analysis demonstrated that eribulin or NUP62 silencing inhibited the proliferative activity of BC cells, evidenced by decreased Ki-67 expression, and also reduced the expressions of the ferroptosis-related indicators NRF2 and GPX4 (Figures 9D and 9E). At the same time, we confirmed that the overexpression of NUP62 can significantly promote tumor growth, and this effect was restored after knockdown of NRF2, confirming that NUP62 affects BC growth in a NRF2-dependent manner (Figures 9C and 9F). We further measured the lipid peroxidation levels and GSH/GSSG ratio in BC tissues derived from each group. Eribulin or NUP62 knockdown significantly increased the lipid peroxidation levels and decreased the GSH/GSSG ratio (Figures 9G and 9H). Meanwhile, the effect of NUP62 on the lipid peroxidation levels and GSH/GSSG ratio is regulated in a NRF2-dependent manner (Figure 9I). These cumulative findings underscore the pivotal role of NUP62 in driving tumorigenesis *in vivo*.

## Discussion

BC remains a leading cause of cancer-related mortality in women globally, with therapeutic resistance and metastatic progression persisting as major barriers to improved clinical outcomes.^3^ While ferroptosis—an iron-dependent form of regulated cell death driven by lipid peroxidation—has emerged as a promising therapeutic target for overcoming BC resistance, the molecular mechanisms governing ferroptosis sensitivity in BC remain incompletely defined.^17^ In this study, we identified NUP62 as a critical oncogenic regulator in BC, which promotes malignant progression by suppressing ferroptosis through a KEAP1-dependent stabilization of NRF2. Additionally, we repurposed eribulin, an FDA-approved chemotherapeutic, as a novel potential NUP62 inhibitor that disrupts this axis to exert antitumor effects. These findings not only expand our understanding of NUP function in BC biology but also provide a mechanistic basis for precision therapies targeting NUP62-high BCs.

NUPs are traditionally known for their roles in nucleocytoplasmic transport, but emerging evidence indicates their involvement in cancer progression through non-canonical functions, such as regulating gene expression and signaling pathways.^4^ Notably, previous studies have linked NUP62 to tumorigenesis in other contexts, such as its association with immune evasion and treatment response in various cancers.^7^ However, its specific role in BC—particularly its mechanistic connection to ferroptosis—has remained unexplored. Our study extends this understanding by demonstrating that NUP62 is significantly upregulated in BC tissues and cell lines, with high expressions correlating with a poor prognosis across multiple clinical endpoints (OS, PFS, PFI, DSS, and DFI). Functional assays confirmed that NUP62 promotes BC cell proliferation, migration, and colony formation, establishing it as an oncogenic driver in BC.

Ferroptosis, an iron-dependent form of cell death driven by lipid peroxidation, has emerged as a promising target for cancer therapy, yet resistance mechanisms—often involving NRF2 activation—limit its clinical efficacy.^18^ NRF2, a master regulator of antioxidant responses, is tightly controlled by KEAP1, which targets NRF2 for ubiquitination and proteasomal degradation under basal conditions.^19^ Our study reveals a novel regulatory mechanism: NUP62 stabilizes NRF2 by competitively binding to KEAP1, thereby disrupting KEAP1-mediated NRF2 degradation. This mechanism is distinct from other known regulators of the KEAP1-NRF2 axis. For example, oxidative stress or electrophilic compounds modify KEAP1 to release NRF2, while other proteins (e.g., p62) sequester KEAP1 to stabilize NRF2.^20^ In contrast, NUP62 directly interacts with KEAP1 without affecting KEAP1 expression, reducing NRF2-KEAP1 binding and inhibiting NRF2 ubiquitination. This competitive interaction represents a unique mode of NRF2 regulation, highlighting NUP62 as a key modulator of redox homeostasis and ferroptosis in BC. Consistent with this, NUP62 knockdown enhances lipid peroxidation, depletes GSH/GSSG ratios, and induces ferroptosis morphological changes, while NUP62 overexpression has the opposite effects. These phenotypes are rescued by NRF2 modulation, confirming that NUP62’s effects on ferroptosis and tumor progression are NRF2 dependent. Together, these data indicate the NUP62-KEAP1-NRF2 axis as a critical determinant of ferroptosis sensitivity in BC.

The identification of small-molecule inhibitors targeting oncogenic pathways is crucial for translating basic discoveries into clinical benefit.^21^ Through virtual screening of FDA-approved drugs, we identified eribulin as a potent NUP62 inhibitor, with >80% inhibitory efficacy against BC cells. Mechanistically, eribulin destabilizes NRF2, increases its ubiquitination, and reduces its nuclear localization—effects that are partially abrogated by NUP62 knockdown, confirming NUP62 dependency. Eribulin is currently used in the treatment of metastatic BC and soft-tissue sarcomas, but its molecular targets remain incompletely defined.^15^^,^^22^ Our study provides a new mechanistic insight: eribulin’s antitumor activity may stem, in part, from its ability to disrupt the NUP62-KEAP1-NRF2 axis, thereby restoring ferroptosis sensitivity and inhibiting malignant phenotypes. Importantly, eribulin’s efficacy is blunted by NUP62 knockdown, suggesting that NUP62 expression levels could serve as a predictive biomarker for eribulin response—an observation with immediate clinical relevance. These findings support the repurposing of eribulin for NUP62-high BCs, potentially enhancing therapeutic precision. Moreover, they provide a rationale for combining eribulin with ferroptosis inducers, as NUP62 inhibition may sensitize tumors to ferroptotic cell death.

In summary, our study identifies NUP62 as a critical oncogene in BC that promotes tumor progression by suppressing ferroptosis through KEAP1-dependent stabilization of NRF2. We further establish eribulin as a novel NUP62 inhibitor that disrupts this axis, supporting its potential use in NUP62-high BCs. These findings not only expand our understanding of NUP function in cancers but also provide a mechanistic basis for improving ferroptosis-based therapies and repurposing existing drugs for precision oncology.

### Limitations of the study

While our study advances understanding of NUP62 in BC, several limitations warrant consideration. First, the *in vivo* experiments were conducted using xenograft models, which lack the complexity of the human tumor microenvironment. Future studies using patient-derived xenografts (PDXs) or transgenic models would strengthen the clinical relevance of our findings. Another limitation is the lack of GST pulldown and domain mapping assays for NUP62-KEAP1 direct interaction, and future studies could fill this gap. Besides, our study lacks direct evidence (e.g., eribulin-NUP62 binding assays) to confirm NUP62 as eribulin’s direct target, and future binding studies could validate this. Finally, while we demonstrate NUP62’s role in BC, its function in other cancer types and its potential association with other signaling pathways (e.g., PI3K/AKT and MAPK) merit exploration.

## Resource availability

### Lead contact

Further information and requests for resources and reagents should be directed to and will be fulfilled by the lead contact, Na Jin (jinnaldszxyy@163.com).

### Materials availability

This study did not generate new unique reagents.

### Data and code availability

• Data: Data generated in this study and reported in this article will be shared by the lead contact upon request.
• Code: This paper does not report any original code.
• Additional Information: Any additional information required to reanalyze the data reported in this paper is available from the lead contact upon request.

## Acknowledgments

This project was supported by grants from the Loudi Science and Technology Bureau (2024) 17 project. This study was funded by the Loudi Science and Technology Bureau (2024) 17 project.

## Author contributions

Conceptualization, N.J. and Y.L.; investigation, N.J. and Y.L.; data curation, Z.Q.; formal analysis, Z.Q., Y.L., and S.C.; supervision, N.J. and W.C.; writing – original draft, Z.Q., J.Y., and R.L.; writing – review & editing, N.J. and Y.L.; visualization, Y.C., X.Y., and S.F.; project administration, N.J.; funding acquisition, Z.Q. All authors have reviewed the manuscript.

## Declaration of interests

The authors declare no competing interests.

## STAR★Methods

### Key resources table

**Table:** 

REAGENT or RESOURCE	SOURCE	IDENTIFIER
**Antibodies**		

β-actin	Proteintech	51067-2-AP; RRID:AB_2086128
NUP62	Proteintech	13916-1-AP; RRID:AB_2267660
NRF2	Proteintech	16396-1-AP; RRID:AB_2782956
SLC7A11	Proteintech	26864-1-AP; RRID:AB_2880661
GPX4	Proteintech	67763-1-Ig; RRID:AB_671515
Ubiquitin	Proteintech	10201-2-AP; RRID:AB_2909469
Histone H3	Proteintech	17168-1-AP; RRID:AB_2716755
Flag	Proteintech	66008-4-Ig; RRID:AB_2918475
HA	Proteintech	81290-1-RR; RRID:AB_2935602
His	Proteintech	66005-1-Ig; RRID:AB_11232599
KEAP1	Proteintech	10503-2-AP; RRID:AB_2132625

**Bacterial and virus strains**		

DH5α Competent cell	Vazyme	Cat#C502-02

**Biological samples**		

Human breast cancer and adjacent normal tissue	Loudi Central Hospital	Ethical official number: 2025- Ethics (Research)-064-01
Animal investigation	the Beijing Keweite Animal Technology Co., Ltd.	Ethical official number: KWT-2025-08-19-02

**Chemicals, peptides, and recombinant proteins**		

NAC	MedChemExpress	HY–K0202
Eribulin	MedChemExpress	HY-13442
Protein A/G Magnetic Beads	MedChemExpress	HY–K0202
Ferrostatin-1	Selleck	S7243
MG-132	Selleck	S2619
Cycloheximide	Selleck	S7418
Lipofectamine 2000	Invitrogen	Cat# 11668019
Trizol reagent	Clontech Laboratories	–

**Critical commercial assays**		

Cell Counting Kit-8	Beyotime	Cat#C0040
GSH/GSSG assay kit	Abcam	ab138881
Liperfluo kit	Dojindo	L248
H_2_DCFDA	Solarbio	#ID3130
GPX4-specific Activity Kit	Elabscience	E-BC-K883-M

**Experimental models: Cell lines**		

HEK293	CCTCSC	GDC0067
MCF10A	CCTCSC	GDC0638
MDA-MB-231	CCTCSC	GDC0297
MCF7	CCTCSC	GDC0055
SK-BR-3	CCTCSC	GDC0286

**Experimental models: Organisms/strains**		

Female nude mice	Beijing	4 weeks

**Oligonucleotides**		

Primers please see the Methods	–	–

**Recombinant DNA**		

pcDNA3.0	Vigene Biosciences	–

**Software and algorithms**		

Fiji	ImageJ	https://fiji.sc
GraphPad Prism	GraphPad Inc.	https://www.graphpad.com

### Experimental model and study participant details

#### Human specimens

Tissues, including 12 pairs of breast cancer and adjacent normal tissues, were collected with informed consent from patients in Loudi Central Hospital. Loudi Central Hospital's Ethics Committee approved this study of patient specimens.

#### Cell lines

All cells involved in our study were purchased from the cell bank of Chinese Academy of Sciences (Shanghai, China). All cells were cultured in complete DMEM, supplemented with 10% FBS and 1% penicillin/streptomycin. The cells were maintained in a 37°C incubator with 5% CO2.

#### Animal models

Female BALB/c nude mice (4 weeks) were purchased from the Beijing Keweite Animal Technology Co., Ltd (Beijing, China). All animal investigations were approved by the Animal Care and Use Committee of the Beijing Keweite Animal Technology Co., Ltd (KWT-2025-08-19-02).

### Method details

#### Cell viability and proliferation assays

Cell viability was quantified using the Cell Counting Kit-8 (CCK-8, Beyotime, Cat#C0040) following the manufacturer's instructions. Briefly, 5×10^3^ cells/well were seeded in 96-well plates and cultured for 12 h. CCK-8 reagent (10% v/v) was added, and plates were incubated at 37°C for 1 h. Absorbance at 450 nm was measured using a Synergy HTX microplate reader (Agilent). For colony-formation assays, 1×10^3^ cells/well were cultured in 6-well plates for 14 days. Colonies were fixed with 4% paraformaldehyde, stained with 0.5% crystal violet, and enumerated microscopically.

#### Reagents

The following chemicals and reagents were used: NAC (HY-B0215), Eribulin (HY-13442) and Protein A/G Magnetic Beads (HY–K0202) from MedChemExpress; Ferrostatin-1 (S7243), MG-132 (S2619) and Cycloheximide (S7418) from Selleck.

#### Plasmid construction and transfection

All overexpression and knockdown plasmids involved in this study were purchased from GenePharma (Shanghai, China). Transfection was performed using Lipofectamine 3000 (Invitrogen) following the manufacturer's protocol, and positive cells were selected with 2 μg/mL puromycin for at least 7 days.

#### RNA isolation and real-time PCR

Total RNA was isolated from tissues or cells using the RNeasy Mini Kit (QIAGEN, Germany), followed by quantification via A260/A280 absorbance ratios. cDNA synthesis was performed with a Promega Reverse Transcription System. qRT-PCR was conducted on an ABI 7500 platform using SYBR Green methods. Relative gene expression, normalized to GAPDH, was calculated using the 2−ΔΔCt method. The primer sequences are listed as follows:

GAPDH: 5′-GGATTTGGTCGTATTGGGCG-3′ (Forward), 5′-ATCGCCCCACTTGATTTTGG-3′ (Reverse);

NUP62: 5′-GGAACAGCGACTCTTGCTTC-3′ (Forward), 5′-GGTGCTCGATATGGCATTAGTG-3′ (Reverse);

SLC7A11: 5′-TCTCCAAAGGAGGTTACCTGC-3′ (Forward), 5′-AGACTCCCCTCAGTAAAGTGAC-3′ (Reverse);

GPX4: 5′-GAGGCAAGACCGAAGTAAACTAC-3′ (Forward), 5′-CCGAACTGGTTACACGGGAA-3′ (Reverse);

FTH1: 5′-CCCCCATTTGTGTGACTTCAT-3′ (Forward), 5′-GCCCGAGGCTTAGCTTTCATT-3′ (Reverse);

GCLM: 5′-TGTCTTGGAATGCACTGTATCTC-3′ (Forward), 5′-CCCAGTAAGGCTGTAAATGCTC-3′ (Reverse);

GCLC: 5′-GGAGGAAACCAAGCGCCAT-3′ (Forward), 5′-CTTGACGGCGTGGTAGATGT-3′ (Reverse);

NQO1: 5′-GAAGAGCACTGATCGTACTGGC-3′ (Forward), 5′-GGATACTGAAAGTTCGCAGGG-3′ (Reverse);

NRF2 (NFE2L2): 5′-TCAGCGACGGAAAGAGTATGA-3′ (Forward), 5′-CCACTGGTTTCTGACTGGATGT-3′ (Reverse).

#### Western blot analysis

Total proteins were isolated from BC tissues or cells, washed twice with PBS, and lysed in RIPA buffer on ice for 30 min. Protein lysates were electrophoretically resolved via SDS-PAGE and transferred to PVDF membranes. Membranes were blocked with 5% BSA for 1h, followed by overnight incubation at 4°C with primary antibodies, including: β-actin (Proteintech, 51067-2-AP, 1:5000), NUP62 (Proteintech, 13916-1-AP, 1:2000), NRF2 (Proteintech, 16396-1-AP, 1:4000), SLC7A11 (Proteintech, 26864-1-AP, 1:2000), GPX4 (Proteintech, 67763-1-Ig, 1:2000), Ubiquitin (Proteintech, 10201-2-AP, 1:4000), Histone H3 (Proteintech, 17168-1-AP, 1:5000), Flag (Proteintech, 66008-4-Ig, 1:1000), HA (Proteintech, 81290-1-RR, 1:1000), His (Proteintech, 66005-1-Ig, 1:5000), KEAP1 (Proteintech, 10503-2-AP, 1:4000). Finally, the membranes were incubated with the corresponding secondary antibodies for 1 h to obtain images.

#### Measurement of GSH/GSSG ratio

The intracellular GSH/GSSG ratio was quantified using a commercial GSH/GSSG assay kit (ab138881, Abcam) following the protocol. Briefly, 1×10^6^ cells were lysed, and the supernatant was collected after centrifugation at 4°C. GSH reacts with DTNB to form the yellow-colored TNB derivative, with its concentration spectrophotometrically determined at 412 nm, where absorbance is proportional to GSH levels.

#### Lipid peroxidation

Lipid peroxides were detected using a Liperfluo kit (Dojindo, L248; Kumamoto, Japan). Cells were incubated with a Liperfluo probe (1μM) at 37°C for 30 min, and then washed with HBSS, and analyzed by flow cytometry or observed under a fluorescence microscope.

#### Reactive oxygen species (ROS) analysis

Cells were enzymatically dissociated, washed twice with PBS, and incubated with 10μM H_2_DCFDA (Solarbio, #ID3130) in serum- and antibiotic-free DMEM at 37°C for 30min. Following PBS washes, intracellular ROS levels were quantified via flow cytometry (FACSCalibur; BD Biosciences).

#### Measurement of GPX4 activity

GPX4 activity was quantified using a GPX4-specific Activity Assay Kit (Elabscience, E-BC-K883-M) following manufacturer's protocol. Briefly, enzymatic reactions were initiated by phosphatidylcholine hydroperoxide addition, and activity was determined by monitoring NADPH oxidation via absorbance decline at 340nm, reflecting glutathione reductase-coupled substrate conversion.

#### Co-immunoprecipitation

Cell lysates were prepared by resuspending pellets in ice-cold IP lysis buffer supplemented with PMSF and protease/phosphatase inhibitors for 15min on ice. Lysates were then centrifuged (12,000×g, 15min, 4°C), and supernatants were transferred for immunoprecipitation. Target proteins were immunoprecipitated overnight at 4°C with gentle agitation using the indicated antibodies. Magnetic beads (Protein A/G, HY–K0202, MCE) were added to capture antibody-antigen complexes. Complexes were washed thrice with ice-cold lysis buffer and eluted by boiling in 2× SDS-PAGE loading buffer (100°C, 10 min).

#### Immunofluorescence assay

BC cells were seeded onto coverslips and allowed to attach overnight. The following day, cells on the coverslips were fixed with 4% paraformaldehyde at room temperature for 15 min, permeabilized in 0.5% Triton X-100 for 15min and blocked with 3% BSA at room temperature for 30 min. The samples were then incubated with the indicated primary antibodies at 4°C overnight. On the next day, the samples were incubated with Goat Anti-Rabbit IgG H&L antibodies or Goat Anti-Mouse IgG H&L antibodies at room temperature for 30 min and washed with TBST three times. Nuclei were counterstained with DAPI, and the slides were visualized under an immunofluorescence microscope (Leica, Germany).

#### Immunohistochemical staining

Formalin-fixed paraffin-embedded (FFPE) tissue sections were heat-adhered at 60°C for 2h. After deparaffinization in xylene and graded ethanol rehydration, antigen retrieval was performed in citrate buffer at 100°C for 30 min. Endogenous peroxidase activity was quenched with 3% H_2_O_2_ in methanol (10 min). Slides were incubated overnight at 4°C with primary antibodies, followed by light-protected incubation with HRP-conjugated goat anti-mouse/rabbit secondary antibodies at room temperature for 1h. Chromogenic detection used 3,3′-diaminobenzidine (DAB), hematoxylin counterstaining, and mounting with synthetic resin.

#### Transmission electron microscopy (TEM)

BC cells precipitates were collected and fixed in an electron microscope fixative at 4°C for 1h. TEM imaging was carried out by Servicebio (Wuhan, China).

#### *In vivo* tumor experiments

For subcutaneous inoculation, 5×10^6^ indicated MDA-MB-231 cells were resuspended in 100μl PBS and implanted subcutaneously into the right flank regions of the mice. Tumor size was measured with calipers every 5 days, and tumor volume was calculated using the formula: volume = length × (width)^2^/2. The mice were sacrificed at the end of the experiments, and the subcutaneous tumors were weighed and photographed.

### Quantification and statistical analysis

All statistical analyses were performed using GraphPad Prism software. Data are presented as mean ± SD from ≥3 independent experiments performed in triplicate (n represents the number of independent biological replicates unless otherwise specified in figure legends). Intergroup comparisons employed unpaired Student's t-tests, while multigroup analyses utilized one-way or two-way ANOVA. Statistical significance was defined as P < 0.05. Specific statistical tests, exact n values, and significance levels for each experiment are provided in the corresponding figure legends and results section.

## Supplemental information

Data S1. Raw western blot data for figures
