# Supplementary material for: NUP62 promotes breast cancer progression and inhibits ferroptosis by stabilizing NRF2 in a KEAP1-dependent way
Source: iScience. 2026 Mar 26;29(5):115484. doi: 10.1016/j.isci.2026.115484 (PMC13092479; doi:10.1016/j.isci.2026.115484)
Supplement: Data S1. Raw western blot data for figures [file mmc1.pdf]

iScience, Volume *29*

Supplemental information

NUP62 promotes breast cancer progression and inhibits ferroptosis by stabilizing NRF2 in a KEAP1-dependent way

Ziran Qiu, Yu Lin, Shanzheng Chen, Wenqing Cao, Jun Yang, Rui Li, Yuan Chen, Xiaoqing Yao, Senlin Fu, and Na Jin

Figure 1B

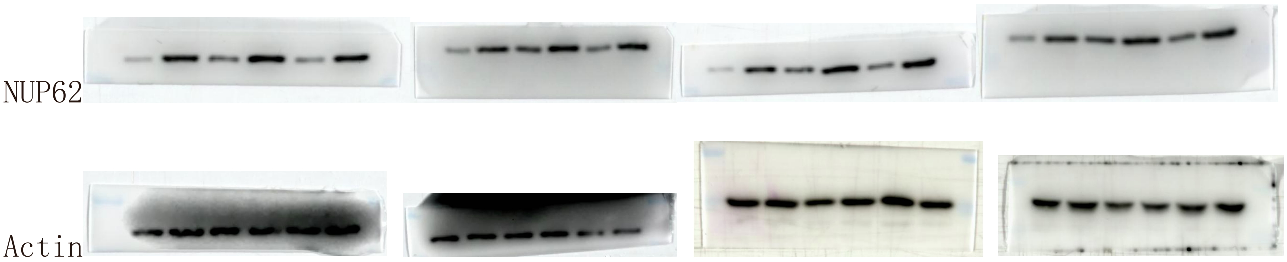

Figure 1G

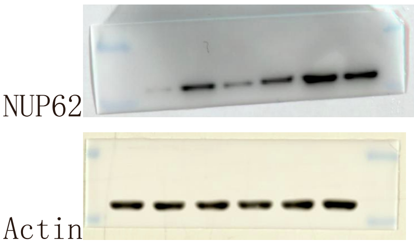

Figure 2B

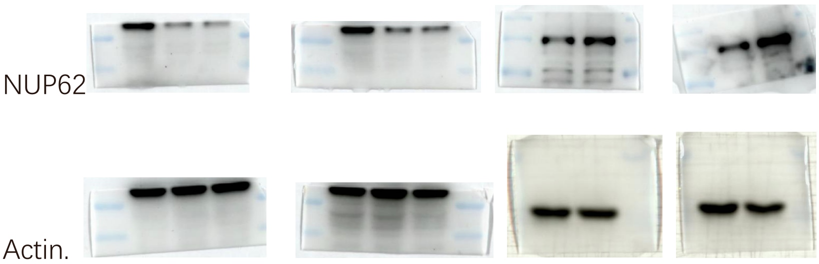

Figure 4F

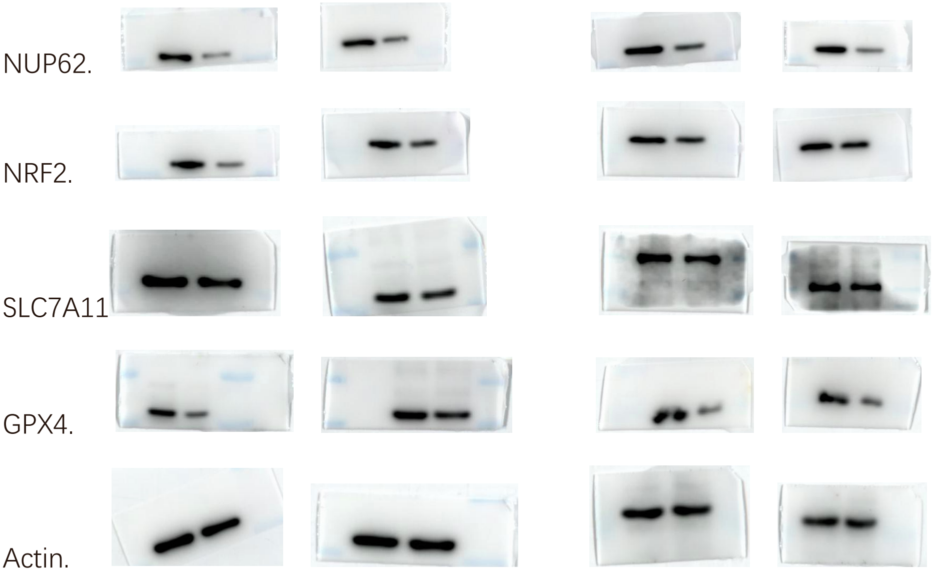

Figure 4G

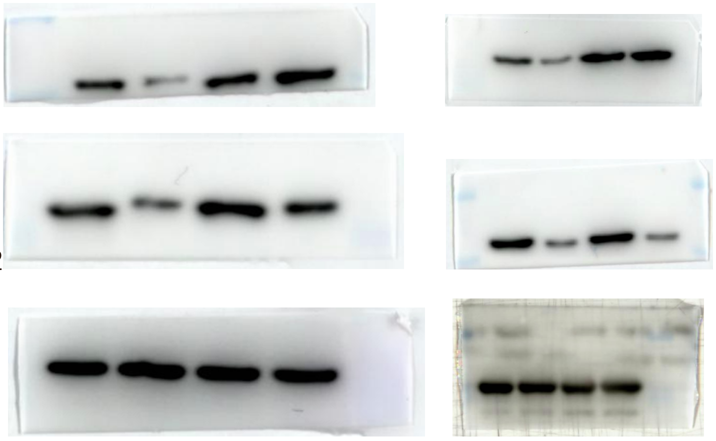

NRF2.

NUP62

Actin

Figure 4H

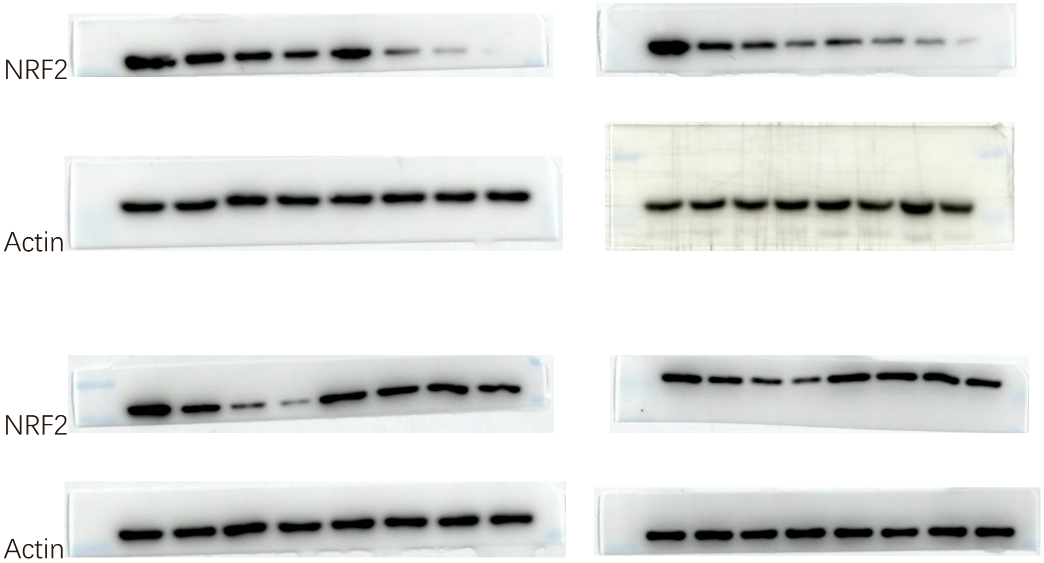

NRF2

Actin

NRF2

Actin

Figure 4I

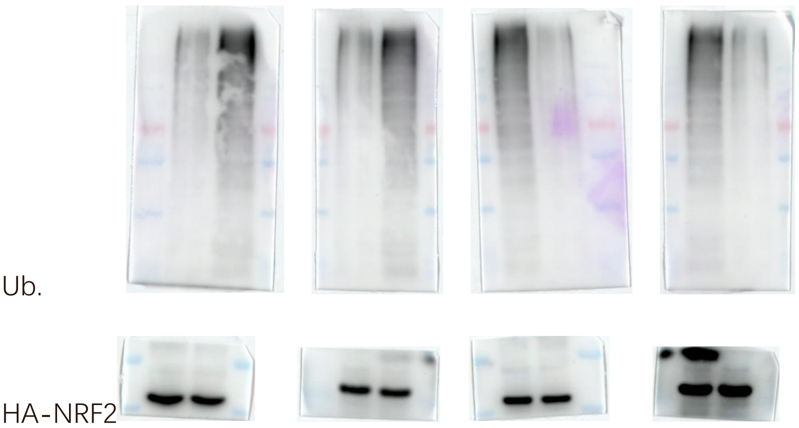

Ub.

HA-NRF2

Figure 4J

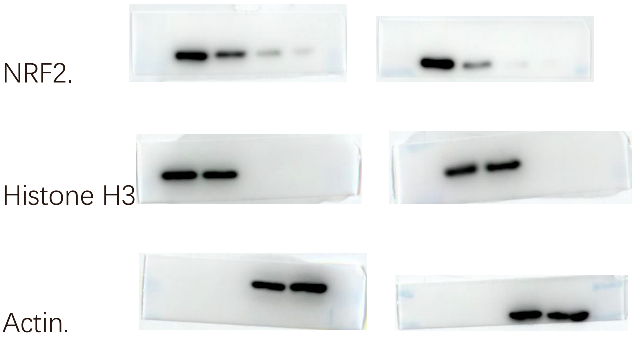

Figure 4K

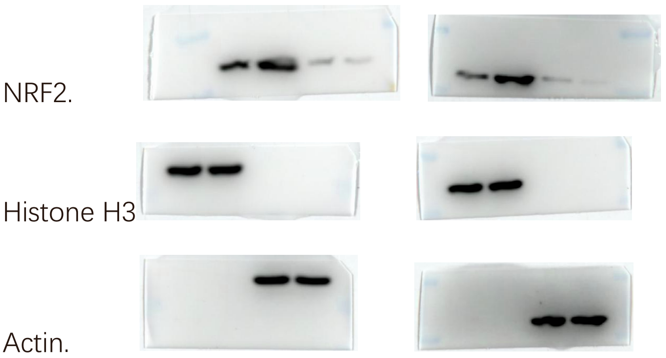

Figure 5A

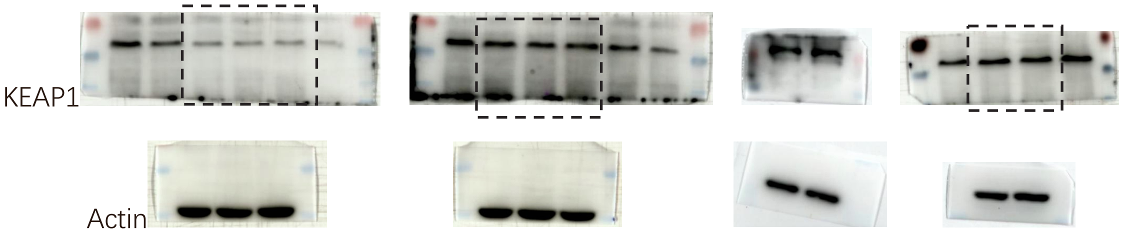

Figure 5C

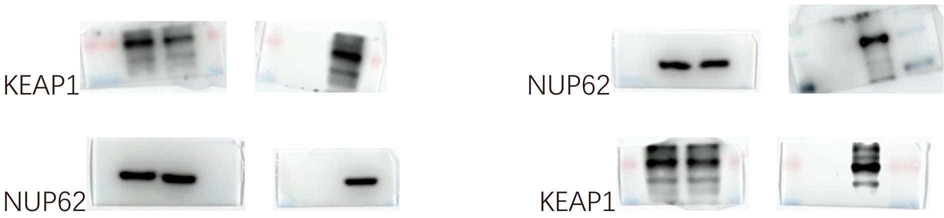

Figure 5D

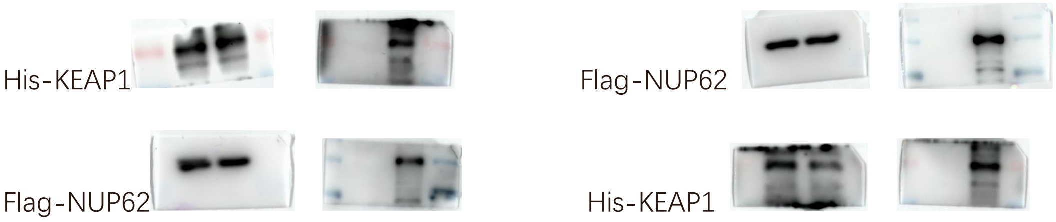

Figure 5F

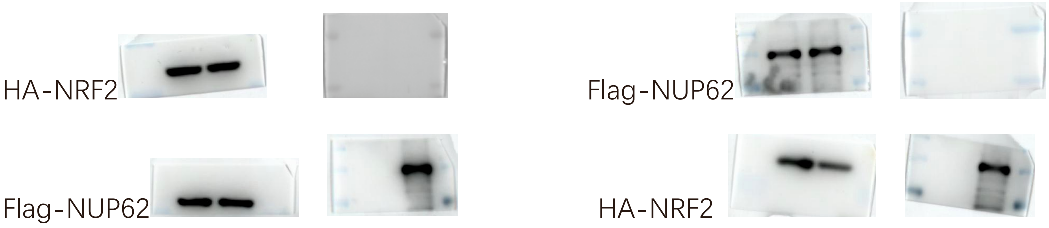

Figure 5G.

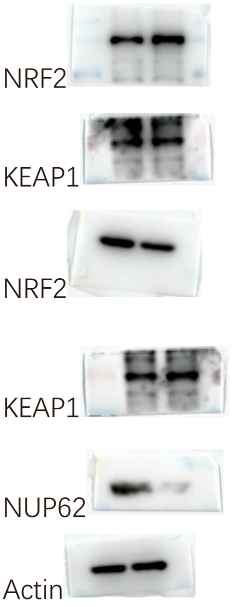

Figure 5H

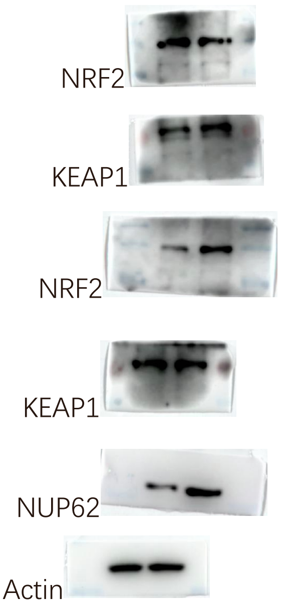

Figure 6A

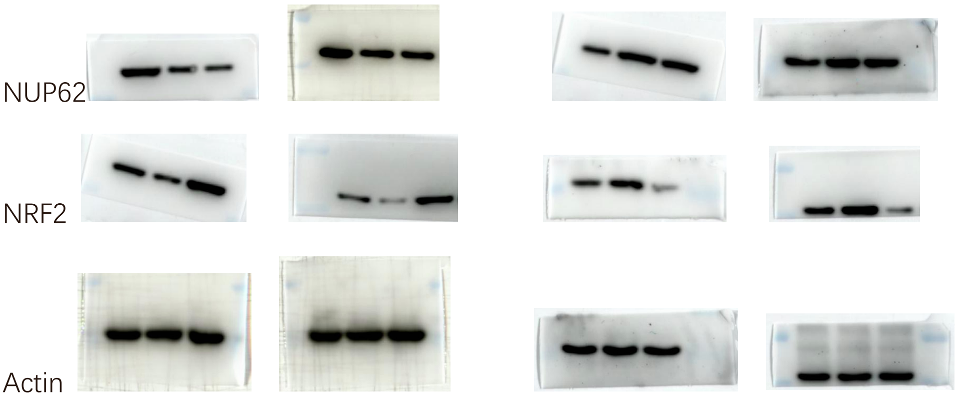

Figure 8A

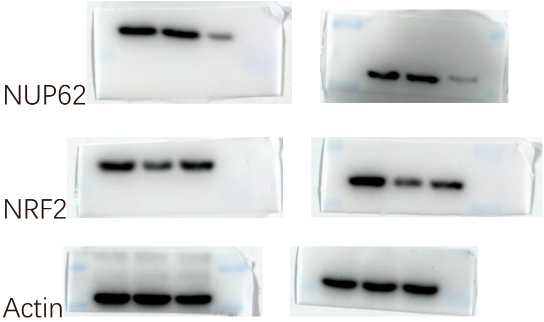

Figure 8B

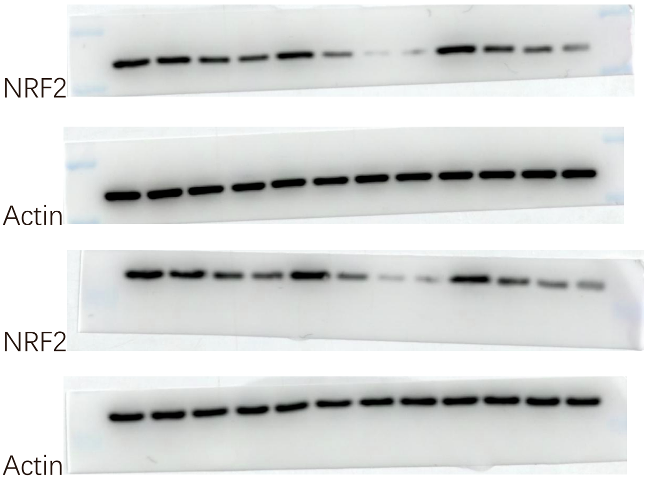

Figure 8C

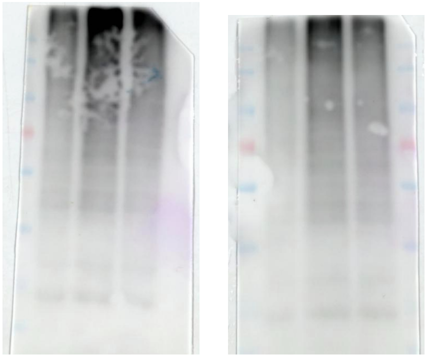

Ub.

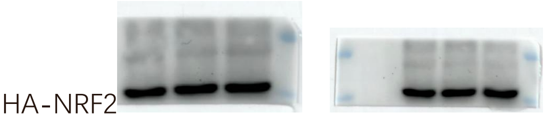

HA-NRF2

Figure 8D

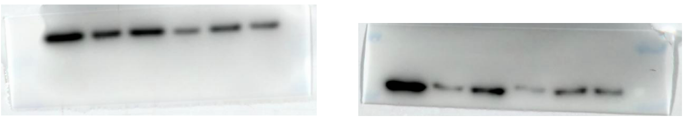

NRF2.

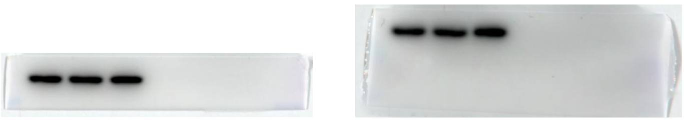

Histone H3

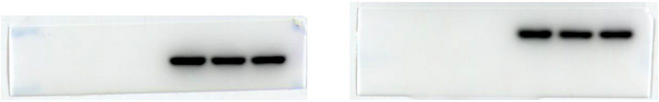

Actin.
